# Genetic variations in drug-induced liver injury (DILI): resolving the puzzle

**DOI:** 10.3389/fgene.2012.00253

**Published:** 2012-11-16

**Authors:** Camilla Stephens, M. Isabel Lucena, Raúl J. Andrade

**Affiliations:** ^1^Unidad de Gestión Clínica de Enfermedades Digestivas, Servicio de Farmacología, Instituto de Investigación Biomédica de Málaga-IBIMA, Hospital Universitario Virgen de la Victoria, Universidad de MálagaMálaga, Spain; ^2^Centro de Investigación Biomédica en Red de Enfermedades Hepáticas y DigestivasBarcelona, Spain

Despite stringent requirements for drug development imposed by regulatory agencies, drug-induced liver injury (DILI) is an increasing health problem and a significant cause for failure to approve drugs, market withdrawal of commercialized medications, and adoption of regulatory measures. The pathogenesis is yet undefined, though the rare occurrence of idiosyncratic DILI (1/100,000–1/10,000) and the fact that hepatotoxicity often recurs after re-exposure to the culprit drug under different environmental conditions strongly points toward a major role for genetic variations in the underlying mechanism and susceptibility. Pharmacogenetic studies in DILI have to a large extent focused on genes involved in drug metabolism, as polymorphisms in these genes may generate increased plasma drug concentrations as well as lower clearance rates when treated with standard medication doses. A range of studies have identified a number of genetic variants in drug metabolism Phase I, II, and III genes, including *cytochrome P450* (CYP) *2E1*, *N-acetyltransferase 2*, *UDP-glucuronosyltransferase 2B7*, *glutathione S-transferase M1*/*T1*, *ABCB11*, and *ABCC2*, that enhance DILI susceptibility (Andrade et al., 2009; Agundez et al., 2011). Several metabolic gene variants, such as *CYP2E1*c1 and *NAT2* slow, have been associated with DILI induced by specific drugs based on individual drug metabolism information. Others, such as *GSTM1* and *T1* null alleles have been associated with enhanced risk of DILI development induced by a large range of drugs. Hence, these variants appear to have a more general role in DILI susceptibility due to their role in reducing the cell's antioxidative capacity (Lucena et al., 2008). Mitochondrial superoxide dismutase (SOD2) and glutathione peroxidase 1 (GPX1) are two additional enzymes involved in combating oxidative stress, with specific genetic variants shown to enhance the risk of developing DILI (Lucena et al., 2010).

Nevertheless, there are discrepancies in the findings and many studies are based on small cohorts and subsequently insufficient to confirm a prominent association between genetic variability in drug metabolism and the risk of DILI. It is important to note that interethnic differences in allele frequency are present and should be considered as a source of heterogeneity when analysing diverse ethnicities. In addition, populations with low variant allele frequencies require high sample sizes to achieve adequate statistical power. Although significant associations between specific drug metabolism gene variants and DILI susceptibility have been found, the low relative risk associated with these variants prevents clinical translation, such as the development of predictive biomarkers (Agundez et al., 2012). Screening for specific risk genotypes identified to date prior to prescriptions would not adequately reduce the number of DILI incidents and could also prevent treatment for many risk allele carriers that would not develop DILI if taking the specific drug.

The introduction of genome-wide association (GWA) studies brought great expectations for the revelation of genetic components responsible for DILI susceptibility. Unfortunately, the outcome has yet not reached the anticipated results. Despite the wide coverage of variants across the entire genome with this technique, none of the previously identified drug metabolizing gene variants has been confirmed. In fact, only specific human leukocyte antigen (HLA) alleles have been significantly associated with idiosyncratic DILI in the GWA studies performed to date (Daly, 2012). The HLA genes are located in the major histocompatibility complex (MHC) region on chromosome 6 and play a prominent role in the human immune system. The class I and II HLA genes encodes for cell surface antigen presenting proteins while the class III derived proteins have variable functions, such as comprising the complement system. The class I and II genes display a large degree of polymorphisms. This variability stems from the need to successfully display a wide range of processed foreign peptides to T cell antigen receptors.

The lack of drug metabolizing gene associations could be due to limitations of the GWA technique as several factors restricting the GWA utility have been identified: (1) very rare genetic variants with very weak effects are not detectable by stringent statistical association analyses, (2) a high number of variants may be present for the same gene with different effects on protein function and the combination of variants may be even more complex in multifactorial diseases, such as DILI, (3) phenotypic diversity in DILI cohorts may “dilute” the presence of specific genetic variations, (4) incomplete coverage of the genome due to technical performance or to variations in linkage disequilibrium strength between single nucleotide polymorphisms (SNPs) in the tested population and the population used to design the array, (5) systemic errors due to differences in variant frequencies between different ethnicities, (6) geographic diversity within the same ethnicity may lead to population-specific effects that, if not accounted for, may prevent identification of disease variants (Karlsen et al., 2010). The last point is particularly important in terms of DILI. Due to the relative rarity of this condition, a single center is unlikely to obtain sufficient cases for successful studies. Collaborative efforts are therefore needed to reach a high number of cases, which can come at the expense of population differences. In addition to array based GWA studies, whole-genome sequencing is fast approaching as a means of searching for genetic variations that contribute to medical conditions. The introduction of “next generation sequencing technologies” has led to a dramatic fall in sequencing cost and together with the increase in genetic information obtained, compared to more conventional array based techniques, whole-genome sequencing offers an enormous potential. Nevertheless, whole-genome sequencing is not without bioinformatic and analytical challenges and its usefulness in personalized medicine will require concerted efforts between multiple groups in a wide range of disciplines (Cordero and Ashley, 2012).

Genetic heterogeneity is common in complex diseases, which points toward a likelihood of multiple genes and pathways being implicated in DILI development. Changes in any of these genes or pathways may lead to the same phenotype, which complicates the identification of specific genetic risk factors. Moreover, the genetic redundancy existing in the human genome may very well compensate for the disruption of a single gene and consequently lead to a neutral effect. A combination of genetic variants affecting several genes simultaneously may therefore be required to produce a specific phenotype or cellular environment in which the presence of an interfering drug compound could lead to DILI. Hence, the importance here is not the individual variants *per se* but the resulting cellular phenotype, which could be reached through various polymorphic combinations. Future studies focusing on cellular/metabolic pathways and variant combinations in well-characterized cohorts are therefore needed. GWA studies have a clear advantage here due to their ability to screen a large number of variants simultaneously. However, the statistical side of this technique is somewhat lagging behind as single polymorphisms are generally considered individually, controlling the overall type I error rate by correcting for multiple testing. Considering biological pathways and variant interactions in GWA study analyses could potentially lead to more meaningful results and is currently being explored (Wang et al., 2010). This could lead us toward the ultimate goal in DILI studies, the identification of genetic risk factors to provide a better insight into the underlying pathogenesis and enable the development of new diagnostic tools along with new safer treatment strategies.
